# A high-fat diet impairs reproduction by decreasing the IL1β level in mice treated at immature stage

**DOI:** 10.1038/s41598-017-00505-0

**Published:** 2017-04-03

**Authors:** Jie Zhang, kai Li, Miao Yuan, Jie Zhang, Guizen Huang, Jie Ao, Haoze Tan, Yanyan Li, Di Gong, Jun Li, lei Kang, Nini An, Fei Li, Ping Lin, Lugang Huang

**Affiliations:** 1 Department of Pediatric Surgery, West China Hospital, Sichuan University, Chengdu, China; 2 Division of Experimental Oncology, State Key Laboratory of Biotherapy, West China Hospital, Sichuan University, and Collaborative Innovation Center for Biotherapy, Chengdu, China; 3 Department of Pediatric Surgery, Provincial People’s Hospital, Guizhou, China

## Abstract

Obesity causes low-grade inflammation that is involved in male infertility. Interleukin 1 beta (IL1β) plays an important role in this process. A high-fat diet (HFD) is the most common cause of obesity. However, the effect of a HFD on IL1β and its consequence in reproduction remain unclear. We established a HFD model in mice treated at immature stage (mice-TIS) and mice treated at mature stage (mice-TMS). Surprisingly, we found that a HFD decreased IL1β levels and was accompanied by an increase in testosterone in mice-TIS, while the reverse results were observed in mice-TMS. In addition, a HFD caused a reduction in testis macrophages and in the expression of inflammasome-related genes and proteins in mice-TIS. Furthermore, we found that IL1β inhibited testosterone secretion through down-regulating the gene expression of P450SCC and P450c17. However, the influence on mice-TIS that were induced by a HFD was recovered by stopping the HFD. In this study, we are the first to report that a HFD impairs the reproductive system by decreasing IL1β and enhancing testosterone levels in mice-TIS, which are different from the effects in mice-TMS. This provides new ideas for the treatment of obesity-induced infertility.

## Introduction

Childhood overweight and obesity, a result of relative overnutrition and a sedentary life style, has become a major health concern in recent decades. According to the WHO, the number of overweight or obese children increased from 32 million globally in 1990 to 42 million in 2013. Statisticians have predicted that this number will increase to approximately 70 million by 2025^1^. Studies of obesity in childhood suggest that it is associated with a wide range of serious health complications and an increased risk of premature onset of illnesses, including insulin resistance, hyperglycaemia, hypertension, dyslipidaemia, type 2 diabetes, cardiovascular disease and behavioural disorders^2, 3^. Without intervention, childhood obesity will likely continue during childhood, adolescence and adulthood.

Obesity as a state of chronic low-grade systemic inflammation has been accepted throughout the world^4–6^. Lee *et al*. reviewed that adipose tissue produces and releases a variety of pro- and anti-inflammatory cytokines, including tumour necrosis factor-α, interleukin1 (IL1), IL6, and others. IL1β is a pro-inflammatory cytokine that stimulates the secretion of multiple cytokines/chemokines, including IL6, IL8, IL10, IL13, MCP-4 and TNFα^7^. It is produced mainly by monocytes and macrophages through the inflammasome pathway. In testes, IL1β is produced by testicular macrophages and/or Leydig cells^7, 8^. Inflammasomes are intracellular multiprotein complexes that sense exogenous and endogenous danger signals through NOD-like receptors, and the intracellular inflammasome complex consists of sensors (Aim2, NLRP3 or NLRC4), an adaptor molecule (ASC), and pro-caspase-1^9^. Inflammasome activation due to multiple types of tissue damage or pathogen-associated signatures results in the autocatalytic cleavage of caspase-1, which plays an important role in the maturation of IL1β and IL18 into active cytokines^10–12^.

Leydig cells are located within the interstitial compartment of the testes and are responsible for the production of androgens and other hormones, including testosterone, androstenedione and dehydroepiandrosterone, which play an important role in normal masculinization, development of the testes, maintenance of spermatogenesis and general male fertility^13–15^. The biosynthesis of testosterone involves four critical enzymes, including a cholesterol side-chain cleavage enzyme (P450SCC), 17α-hydroxylase/C17-20 lyase (P45017α), 3-hydroxysteroid dehydrogenase/Δ5–Δ4 isomerase (3βHSD), and 17-ketosteroid reductase^16^.

A scheduled apoptotic wave of germ cells is necessary for normal mature spermatogenesis^17–19^. During normal spermatogenesis, apoptosis is a sporadic event, occurring mainly among spermatogonia. An early and massive wave of germ cell apoptosis occurs in testes between 2 and 4 weeks after birth. It has been proposed that the early wave of apoptosis is necessary for the maintenance of a critical ratio of cells of some germ cell stages^18^.

The harm to male fertility caused by high-fat diet (HFD)-induced obesity has been widely discussed^20–22^. Much research has found that a HFD is associated with high plasma IL1β levels^4, 23, 24^. Chronic overproduction of IL1β has been linked to multiple immune diseases, including type 2 diabetes and its macrovascular complications^25^. In addition, IL1β is also known to modulate Leydig cell testosterone production^26–28^. However, little is known about the role of IL1β in the reproductive system of overweight or obese children and the mechanism of HFD-induced changes in the IL1β level in testes. Moreover, many studies have focused on the relationship between a HFD and the hypothalamic-pituitary–gonad axis and testes; the direct and partial influences of a HFD at different ages on the testes have rarely been reported. By establishing several HFD mouse models, we observed the effects of a HFD on the development of testes in mice-TIS. Testosterone and germ cell apoptosis were assessed in mice given different HFD regimens. IL1β and the number of macrophages in the testes of HFD-fed both mice-TIS and mice-TMS were measured to try to explain the phenomenon. In addition, by using *in vitro* experiments with isolated testes, we excluded the involvement of the hypothalamus and the pituitary gland and confirmed the direct effects of IL1β on Leydig cells. We also tested the expression of inflammasome-related genes and proteins to examine whether the inflammasome pathway plays a role in IL1β production in the testes of mice fed a HFD. Furthermore, the effect of IL1β on the testosterone production of Leydig cells was confirmed both *in vivo* and *in vitro*. We also observed the restoration of HFD mice testes after stopping the HFD.

## Results

### HFD-feeding increases secretion of testosterone and inhibits apoptosis of germ cells in mice-TIS but has the opposite effect in mice-TMS

**Figure 1:**
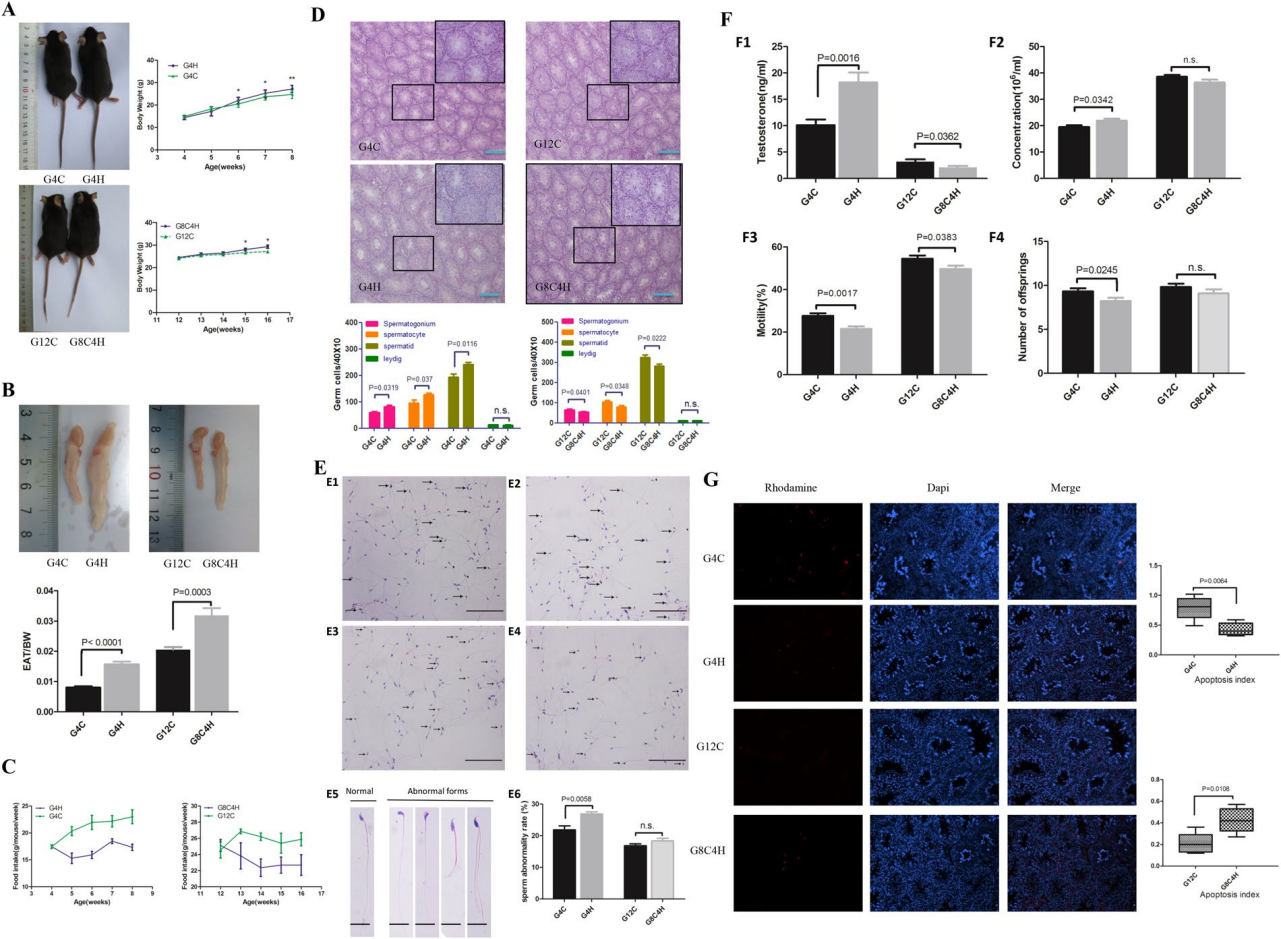
Description of mouse model established after mice were fed a HFD for 4 weeks and the influence of a HFD on IL1β in the reproductive system of mice. (**A**) Representative pictures of G4H, G4C, G8C4H and G12C mice and comparison of time-dependent increases in bodyweight between G4H and G4C, G8C4H and G12C mice (n = 10 in each group). (**B**) Representative picture of epididymal adipose tissue and the epididymal adipose tissue to bodyweight ratio (EAT/BW) in the G4H and G8C4H groups compared to the control group. (**C**) Food intake in G4H (left) and G8C4H (right) mice compared with the corresponding control group. (**D**) Haematoxylin and eosin-stained mice testis sections from each group and the germ cell count. Scale bars = 50 μm. (**E**) Sperm morphology analysis of G4C (E1), G4H (E2), G12C (E3) and G8C4H (E4) mice using HE staining. Arrows indicate abnormal spermatozoa. Scale bar = 50 μm. (E5) Normal and abnormal spermatozoa in different forms; Scale bar = 10 μm. (E6) Comparison of sperm abnormality in mice fed either a HFD or a CD. (**F**) Testosterone level (F1), sperm concentration (F2), sperm mobility (F3) and the number of offspring (F4) in G4H and G8C4H mice compared to G4C and G12C mice. (**G**) Germ cell apoptosis was tested use a TUNEL assay in G4H and G8C4H mice compared to G4C and G12C mice, respectively. Apoptotic germ cells are stained red (Rhodamine), and blue represents DAPI-stained nuclei. Merged images were produced using Image J software. Percentage of TUNEL-positive germ cell values are expressed as the mean ± SEM. *P < 0.05, **P < 0.01.

To observe the effect of a HFD on the mice in different developmental periods, a HFD was given to immature (<7–9 weeks) and mature mice (>9 weeks)^29^, termed the G4H and G8C4H groups, as described in Materials and Methods. As expected, the bodyweight (BW) and the ratio of epididymal adipose tissue (EAT)/bodyweight were significantly elevated in G4H and G8C4H groups compared to G4C and G12C groups (Fig. 1A,B), whereas both G4H and G8C4H mice had lower food intake compared to G4C and G12C mice (Fig. 1C). After HE staining of testes, the number of germ cells, including spermatogonia, spermatocytes and spermatids, in testes were counted to assess the influence of a HFD on mouse reproduction. We found that the number of spermatogonia, spermatocytes and spermatids of G4H mice was significantly increased compared with G4C mice. In addition, the number of Leydig cells was not different between G4H and G4C mice (Fig. 1D). However, we observed that the number of spermatogonia, spermatocytes and spermatids of G8C4H mice were decreased compared with G12C mice. As with G4H and G4C mice, there was no difference in the number of Leydig cells of G8C4H and G12C mice (Fig. 1D). Moreover, we also observed that there was a higher ratio of teratosperm in the G4H group compared to the G4C group. However, the ratio of teratosperm have no difference between G12C and G8C4H (Fig. 1E). This suggests that a HFD has a different influence on the spermatogenic cells of mice-TIS and mice-TMS, which is different from normal spermatogenesis. Surprisingly, we found that serum testosterone levels of G4H mice were higher than those of G4C mice. In contrast, the testosterone levels of G8C4H mice were lower than those of G12C mice. In addition, germ cell extracted from the cauda epididymidis revealed that G4H have higher sperm concentration and lower motility than G4C, However, both sperm concentration and motility decreased in G8C4H compared to G12C. Through natural mating assay, we found that the number of offspring of G4H significantly decreased compared to G4C. However, there was no difference in the number of pups per litter between G12C and G8C4H (Fig. 1F). This suggests that a HFD has different effects on the testosterone level and fertility in mice-TIS and mice-TMS.

It is known that testosterone is essential for regulation of spermatogenesis^30^. Because apoptosis of germ cells is an important event in spermatogenesis, the rate of germ cell apoptosis was measured by TUNEL staining. The apoptosis index (AI) was calculated to represent the apoptosis status of each group. Consistent with the serum testosterone findings, the AI was significantly lower in the G4H group compared to the G4C group, but higher in G8C4H mice compared to G12C mice (Fig. 1G). These results imply that a high testosterone level in mice-TIS may induce germ cells to avoid apoptosis.

### IL1β and the number of macrophages are different in mice-TIS and mice-TMS fed a HFD

**Figure 2:**
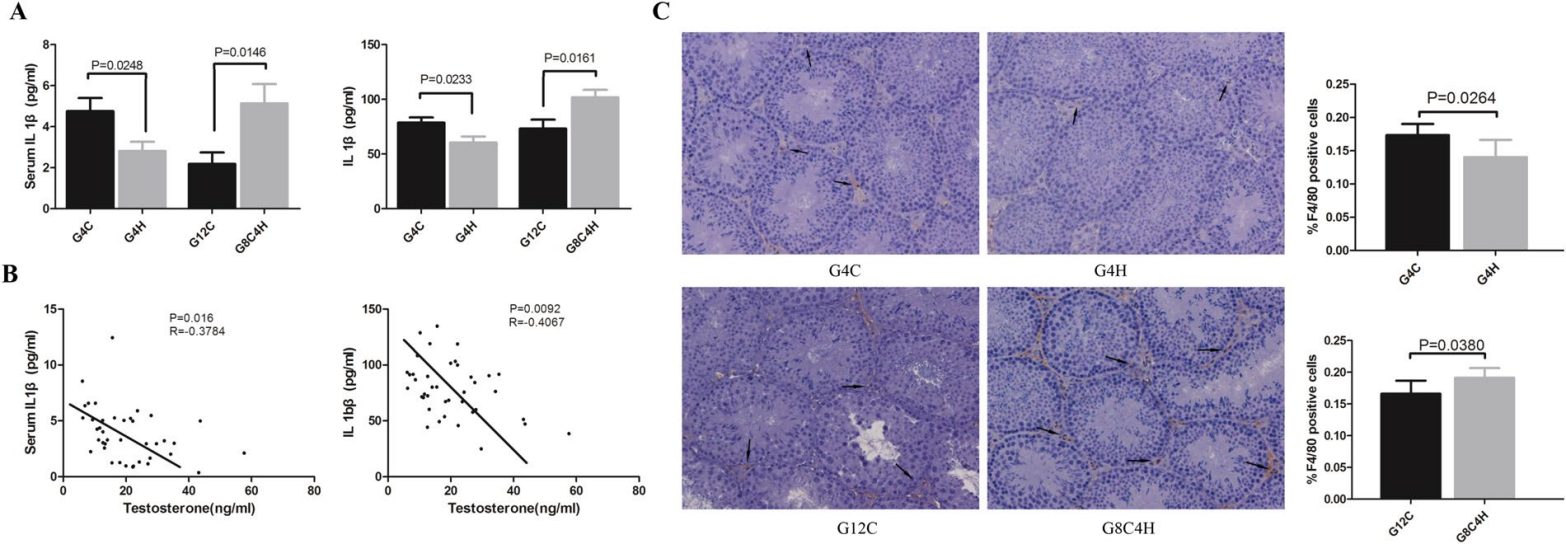
IL1β is negatively correlated with testosterone and the number of macrophages in testes of mice-TIS and mice-TMS. (**A**) Serum and testis IL1β (ELISA) in G4H and G8C4H groups compared with their control group. (**B**) Correlation analysis demonstrated that both serum (left) and testis (right) IL1β levels were negatively correlated with testosterone in mice. (**C**) Immunohistochemical detection of the macrophage-specific antigen F4/80 (black arrows) in testes from each group of mice and comparison of the F4/80-positive cell ratio between G4H and G4C, G8C4H and G12C mice. Scale bars = 100 μm.

Generally, pro-inflammatory IL1β is thought to circulate at a high level in obese people. To understand the IL1β situation in these models, the IL1β in the serum and testis homogenates were measured by ELISA. The results showed that IL1β both in serum and testes of G4H mice was lower compared to G4C mice. However, the IL1β levels in the serum and testes of G8C4H mice were significantly increased (Fig. 2A). Moreover, we found that the IL1β level, either in serum or testis homogenates, was negatively correlated with testosterone level in all experimental mice (Fig. 2B). These results indicate that a HFD has different influences on IL1β in mice-TIS and mice-TMS, and the IL1β level may have a negative correlation with testosterone level in mice.

Macrophages in the testes are the main source of IL1β in testes. To determine the relationship between the change in IL1β and the number of testicular macrophages, F4/80 antigen, a specific marker of macrophages, was tested in testis sections using immunohistochemical staining. We found that the number of macrophages was significantly lower in G4H mice compared to G4C mice. In contrast, it was higher in G8C4H mice compared to G12C mice (Fig. 2C), suggesting that a HFD also influences testicular macrophages, and its effect is different in mice-TIS and mice-TMS. The macrophages may cause the variation tendency in IL1β in testes.

### A HFD inhibits inflammasome activation in testes of mice-TIS but activates the inflammasome in mice-TMS

**Figure 3:**
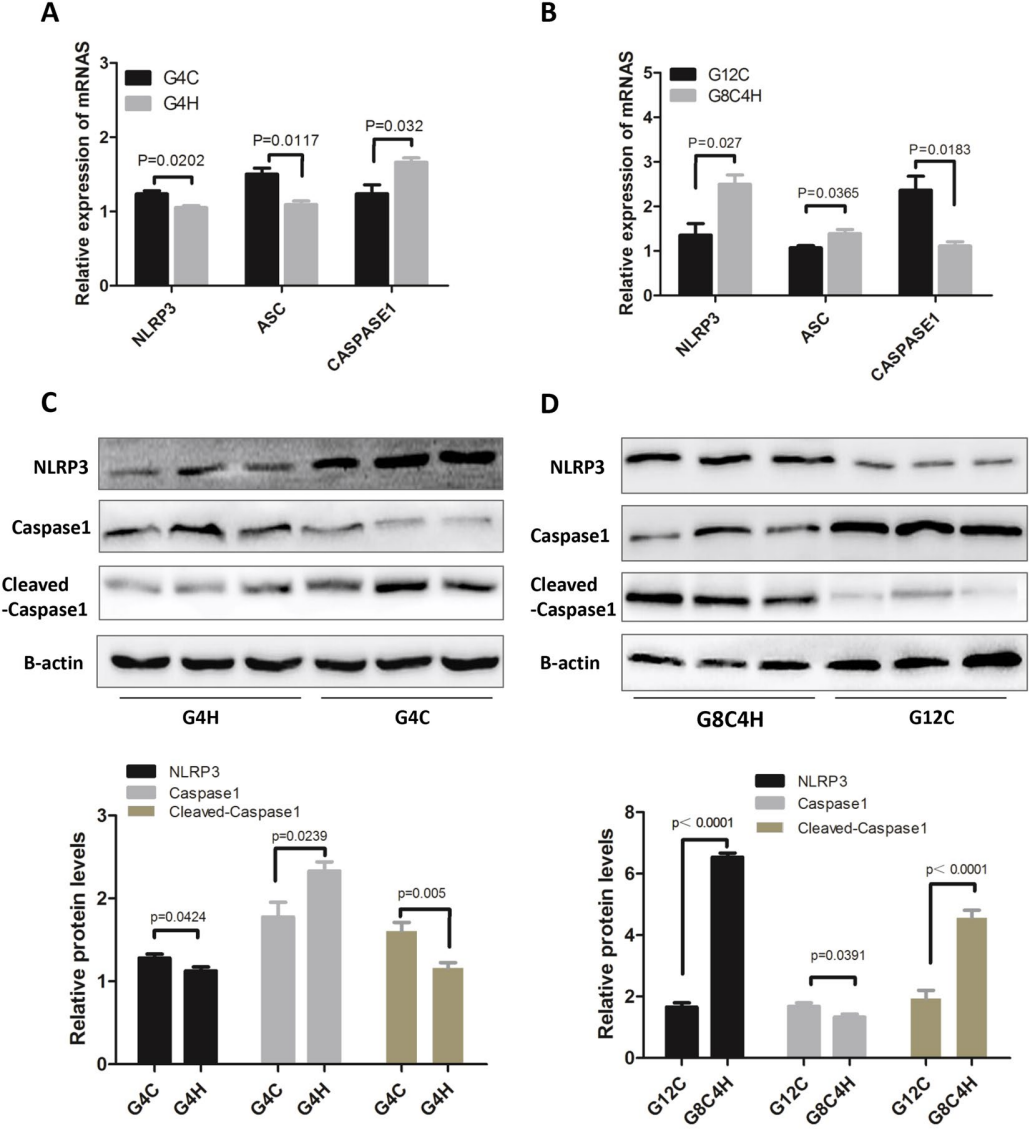
Inflammasome expression in immature and mature mice after fed a HFD. Gene expression of the inflammasome components NLRP3, ASC and caspase-1 were measured in testes using qRT-PCR in G4H and G4C mice (**A**) and in G8C4H and G12C mice (**B**). Total testis protein was extracted from each group: G4H and G4C (**C**), G8C4H and G12C (**D**). Then, western blotting was used to measure the protein expression of NLRP3, caspase-1, cleaved-caspase-1 and β-actin. Western blot analysis of inflammasome-related proteins in the different groups is shown under the corresponding WB results. Densitometric ratio analyses (NLRP3, ASC, caspase-1/β-actin) were performed using Image J software. *P < 0.05 and **P < 0.01.

The inflammasome is the main pathway control maturation and secretion of IL1β and IL18. NLRP3 and caspase-1 activation are the main steps leading to release of IL1β^9, 31, 32^. To clarify the mechanism of the HFD-induced change in IL1β, we tested the mRNA expression of inflammasome-related genes, including NLRP3, ASC and caspase-1 in testes by qRT-PCR. As shown in Fig. 3A, the expression of NLRP3 and ASC were significantly decreased in G4H mice compared to G4C mice. In addition, the expression of caspase1 was increased in G4H compared to G4C mice. However, the gene expression of NLRP3 and ASC were significantly elevated in G8C4H mice compared G12C mice, on the contrary, the expression of caspase1 decreased in G8C4H compared G12C mice (Fig. 3B). To confirm the mRNA results, we next assessed the expression of NLRP3, caspase-1 and cleaved-caspase-1 protein by western blot assay. Indeed, the expression of NLRP3 and cleaved-caspase-1 were decreased in G4H mice compared to G4C mice (Fig. 3C), while they were significantly increased in G8C4H mice compared to G12C mice (Fig. 3D). The trend was consistent with IL1β in mice from the same feed groups. These findings suggest that a HFD may inhibit inflammasomes in mice-TIS but activate inflammasomes in mice-TMS and further affect the production of IL1β.

### IL1β inhibits testosterone synthesis through decreased expression of CYP11A1 and CYP17A1

**Figure 4:**
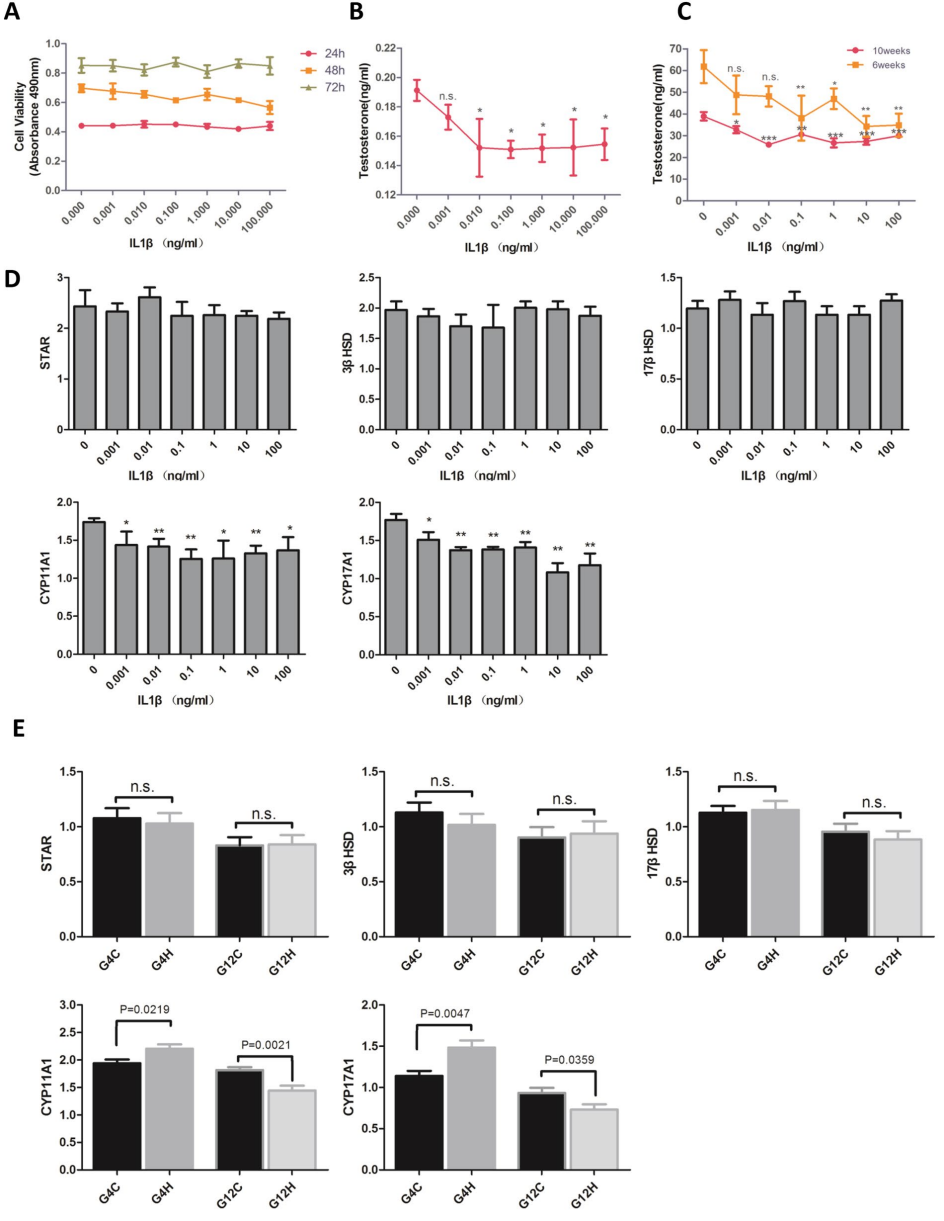
IL1β inhibits the testosterone synthesis of Leydig cells. (**A**) The TM3 cell number stimulated by different concentrations of IL1β for 24 h, 48 h and 72 h. (**B**) Testosterone produced by TM3 cells under the stimulation of different concentrations of IL1β. (**C**) Equal decapsulated testes from 6-week-old mice and 10-week-old mice were incubated with several concentrations of IL1β for 24 h *in vitro*. The testosterone of the supernatants was tested using ELISA. (**D**) The mRNA expression of STAR, P450SCC, P45017α, 3-βHSD and 17β-HSD in TM3 cells treated with different concentrations of IL1β for 24 h. The photograph shows a representative result of three independent experiments. (**E**) The mRNA expression of STAR, P450SCC, P45017α, 3-βHSD and 17β-HSD in G4H, G4C, G12H and G12C testis. *P < 0.05 and **P < 0.01.

Similar to the above results, *in vivo* experiments showed that the testosterone level was negatively correlated with IL1β in mice. However, production of testosterone is influenced by pituitary hormones. To determine whether IL1β directly affects Leydig cells, an MTT assay was performed to detect cell viability of TM3 cells treated with different concentrations of IL1β. In Fig. 4A, the results showed that cell viability was not different in relation to the supply of IL1β. This confirms that IL1β does not affect the survival of Leydig cells, and therefore, we hypothesized that IL1β may inhibit testosterone biosynthesis. To verify this hypothesis, we used IL1β to treat TM3 cells and used an ELISA to test the testosterone of the supernatant. We found that the testosterone level was decreased by IL1β (Fig. 4B). To further confirm the effects of IL1β on testosterone secretion, testicular tissues from 6- and 10-week-old mice were incubated and stimulated with IL1β *in vitro*. Similar with the TM3 cells, testosterone secretion of both mice-TIS and mice-TMS testis tissues were decreased by the treatment with IL1β (Fig. 4C). These results suggest that IL1β directly inhibits testosterone secretion.

To illustrate the mechanism of IL1β inhibition of the production of testosterone in Leydig cells, mRNA was extracted from TM3 cells stimulated with different concentrations of IL1β, and the expression of key enzyme genes for testosterone synthesis were detected by qRT-PCR. We observed no significant differences in the expression of STAR, 3ΒHSD and 17βHSD. However, the gene expression of CYP11A1 and CYP17A1 were significantly reduced after treatment with IL1β (Fig. 4D). *In vivo*, the expression of key enzyme genes for testosterone synthesis in testis from G4H, G4C, G12H and G12C have been tested. Similar with the TM3 cells *in vitro*, the expression of STAR, 3ΒHSD and 17βHSD have no significant differences in the paired G4H/G4C, G12H/G12C. However, the gene expression of CYP11A1 and CYP17A1 were increased in G4H compare to G4C and decreased in G12H compared to G12C. Since G4H and G12C have lower IL1β levels compared to its control group (Fig. 4E). Correlation analysis on serum IL1β levels and the mRNA expression of these genes in all experimental mice found that IL1β and the expression of CYP11A1 and CYP17A1 were negatively correlated (Supplementary Figure S2). These results suggest that IL1β inhibits P450SCC and P450c17 gene expression in Leydig cells, which then inhibits testosterone production.

### The detrimental effects of a HFD on the reproductive system of mice-TIS can be recovered after stopping a HFD

**Figure 5:**
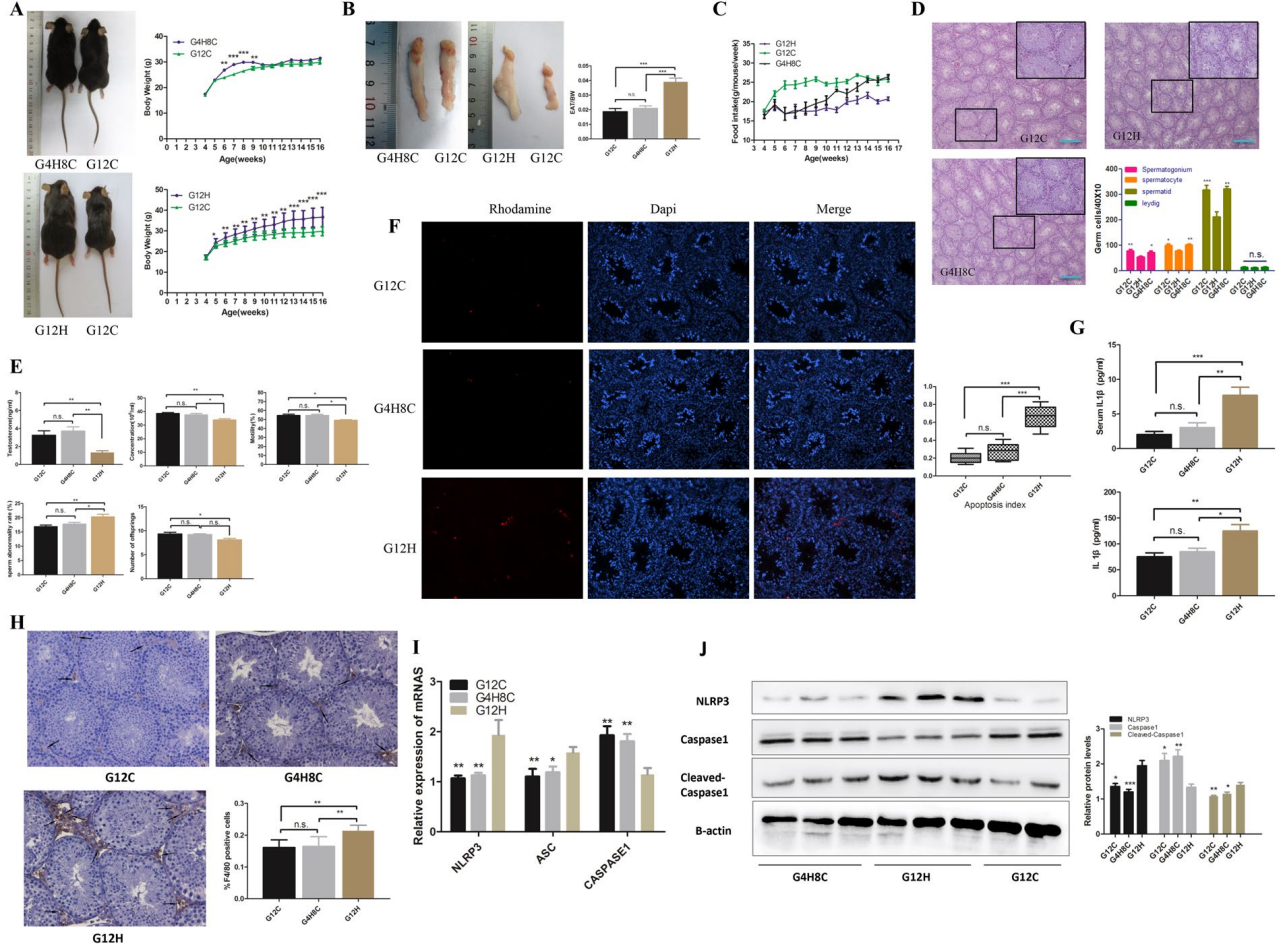
Influence of a HFD on the reproductive system of mice can be recovered by stopping the HFD. (**A**) Representative picture of G8C4H, G12H and G12C mice. Comparison of time-dependent increases in bodyweight between G4H8C, G12H and G12C mice (n = 10 in each group). (**B**) Epididymal adipose tissue and EAT/BW of G4H8C, G12H and G12C mice. (**C**) Food intake in three groups. (**D**) Haematoxylin and eosin-stained testicular sections from G4H8C, G12H and G12C mice. Scale bars = 50 μm. (**E**) Testosterone levels, Spermatic parameters, including sperm concentration, mobility and rate of abnormal sperm, and the number of offspring in each group. (**F**) Germ cell apoptosis was tested using a TUNEL assay in three groups, and the apoptosis index was determined. (**G**) Serum and testis homogenates tested for IL1β (ELISA). (**H**) Immunohistochemical detection of the macrophage-specific antigen F4/80 (black arrows) in testes from G4H8C, G12H and G12C mice and the ratio positive cell between these groups. (**I**) Inflammasome-related gene expression in these three groups (*P < 0.05 compared with the G12H mice group). (**J**) Inflammasome protein expression in these three groups. Data are expressed as the mean ± SEM. *P < 0.05, **P < 0.01. ***P < 0.001; n.s. non-significant difference.

To further investigate whether the effect of a HFD on the reproductive system of mice-TIS is permanent, we established the G12H, G12C and G4H8C mouse models described in Materials and Methods. We found that the bodyweight of G4H8C mice was significantly increased during the HFD period; however, it started to lose weight after being fed a CD. Finally, no difference in bodyweight was observed between G4H8C and G12C mice. In contrast, G12H mice gained significantly more weight than G12C mice (Fig. 5A). We also found that EAT/BW values were significantly higher in the G12H group compared to the G12C group. However, the EAT/BW values of G4H8C mice were not different compared to G12C mice (Fig. 5B). In addition, the food intake of G4H8C and G12H mice was lower than for G12C mice (Fig. 5C). This suggests that the G12H HFD model had been established, and G4H8C mice have a close to normal situation after stopping the HFD for 8 weeks.

HE staining of testes indicated no difference between G4H8C and G12C mice in the number of spermatogonia, spermatocytes and spermatids, but the number of spermatogonia, spermatocytes and spermatids of G12H mice were significantly decreased compared to G12C mice. The number of Leydig cells in the three groups was not different. In addition, we found seminiferous epithelia in G12H mice that were severely disorganized and atrophic, and the cell adhesion between spermatogenic cells and Sertoli cells was disrupted and loosely arranged, while the structure of the seminiferous tubules of G4H8C mice was close to that of G12C mice (Fig. 5D). These data indicate that a HFD impairs the reproductive system through a decrease in the number of germ cells and disordering of germ cells in seminiferous tubules. However, G4H8C mice maintained normal testis structure by stopping the HFD, suggesting the impaired testis structure may recover by removing the HFD.

Moreover, no significant differences in testosterone, sperm concentration, motility and offspring were found between G4H8C and G12C mice, but the parameters listed above were significantly decreased in G12H compared to G12C mice. Besides, there was a higher ratio of teratosperm in the G12H group compared to the G12C and G4H8C group, the ratio of abnormal sperm have no difference between G12C and G4H8C (Fig. 5E). Similarly, the testicular apoptosis index displayed no difference between G4H8C and G12C mice, whereas in G12H mice, the AI were still significantly elevated compared to G12C mice (Fig. 5F). This demonstrated that a continuous HFD is harmful for the reproductive system of mice, and the influence of a HFD on mice-TIS can be recovered after stopping the HFD. In addition, no significant differences in IL1β were observed in the serum and testes between G4H8C and G12C mice. In contrast, IL1β was significantly increased in the serum and testes of G12H mice (Fig. 5G). This suggests that weight loss can recover the IL1β level of G4H mice, and long-term HFD feeding can increase IL1β in mice. Furthermore, we found no significant difference in the number of macrophages between G4H8C and G12C mice. In contrast, macrophages were significantly elevated in G12H compared to G12C mice (Fig. 5H). We also observed no difference in the expression level of inflammasome-related genes in G4H8C mice compared to G12C mice, but the expression of NLRP3 and ASC was dramatically elevated in G12H mice compared to G12C mice (Fig. 5I). The protein expression of NLRP3 and cleaved-caspase-1 was significantly increased in G12H mice compared to G12C mice, while there was no significant difference in the expression of these proteins in G4H8C mice compared to G12C mice (Fig. 5J). These results demonstrated that the changes in inflammation induced with a HFD in mice-TIS can be restored by removing the HFD. In any case, a long-term HFD enhances inflammatory damage.

## Discussion

This is the first report presenting fundamental research investigating the change in IL1β levels in immature mice fed a HFD and the mechanism. Although obesity-induced low inflammatory status is a result of a combination of multiple factors, our study provides a partial basis for the role of IL1β in obesity and reproduction. We established several HFD mouse models to explore the answer. We found that IL1β was decreased in immature mice after being fed a HFD for 4 weeks, but they had a higher testosterone level than the control mice, and a HFD influenced IL1β through inflammasomes in immature mice. In addition, the impairment induced by a HFD in the reproductive system of mice-TIS can be recovered after stopping the HFD accompanied by weight loss. However, the HFD increased the IL1β level and decreased the testosterone level in mice-TMS, and the reason for this IL1β increase was inflammasome activation. Furthermore, we confirmed that IL1β inhibited testosterone secretion.

That the circulating level of IL1β is found to be elevated with obesity/HFD has been reported elsewhere^33, 34^. However, these previous studies focused on the influence of a HFD on mature mice or worked through the hypothalamic–pituitary –gonad axis^35, 36^. Surprisingly, we found that the IL1β level both in serum and the testes was decreased in HFD-fed mice-TIS, but the testosterone level was significantly increased compared to the control group. This is an interesting phenomenon because some scholars report that inflammation is increased with the duration of the HFD or at different time points. Initially, it does not promote inflammation, and then, after 10 weeks, it starts to induce inflammation and then resolves in later ages. Regardless, consistent with other reports, a HFD increased IL1β and decreased testosterone in mice-TMS that were fed a HFD for the same amount of time^23^. After test the testosterone levels of control diet mice in different ages, we found testosterone and age were positively correlated before 8 weeks old and decreasing with age after that (Supplementary Figure S1). This implies that a HFD has different influences on the production of IL1β in mice-TIS and mice-TMS. Wang Y *et al*. found that obesity is associated with early sexual maturation in both boys and girls^22^. Similarly, one study shows that in overweight boys pubertal development begins and reaches the late stage earlier in comparison with normal-weight children^37^. In addition, numerous studies have shown that a HFD or obesity in mature humans and mice leads to low testosterone^20, 35, 38, 39^. Our data shows that immature began treated mice have a higher level of testosterone and sperm concentration, but a lower sperm motility and offspring. Therefore, the high level of testosterone may have a harmful influence on the immature reproductive system. It has been shown that spontaneous germ cell apoptosis occurs during the process of germ cell differentiation in testes, which is an important mechanism to remove abnormal and surplus germ cells, and germ cell apoptosis is under endocrine control^40^. Moreover, spermatogenesis is completely dependent on the pituitary hormone follicle-stimulating hormone and androgen, which regulate spermatogonia proliferation and germ cell development^41–43^. Rodriguez *et al*. found that testosterone injection partly inhibited the early germ cell apoptotic wave and led to altered spermatogenesis^44^. Our data show that the apoptosis index was significantly decreased in HFD-fed mice-TIS testes compared to the control group, which is similar to these reports. At the same time, the ratio of teratosperm increased. Taken together, we provide evidence that a HFD decreased the IL1β level in mice-TIS and induced a high serum testosterone level, which reduced the physiologic germ cell apoptosis and increased the ratio of abnormal sperm that may be closely related to infertility.

Several studies have shown that natural weight loss through diet and/or exercise, the use of drugs such as aromatase inhibitors and surgical methods such as scrotal lipectomy have an effect in treating obesity-related infertility in humans^45–47^. Here, we have explored whether the changes in mice-TIS production will recover after loss of weight by feeding a CD. We have shown that IL1β, testosterone, spermatic parameters, the number of offspring and the apoptosis index of immature mice fed a HFD for 4 weeks and then given 8 weeks of a CD were close to those of the normal control mice. In addition, the testis structure and germ cell count recovered to normal levels compared to G4H8C and G12C mice. This implies that stopping a HFD and weight loss might be an obvious treatment to improve obesity-linked early sexual maturation in children.

Many studies have reported that a HFD activates inflammasomes in mice^24, 48^. Interestingly, we found that a HFD decreased the gene expression levels of NLRP3 and ASC and inflammasome-related proteins, including NLRP3 and cleaved-caspase-1, in immature mice that were fed a HFD for 4 weeks. However, these genes and proteins were increased in mature mice after being fed a HFD, regardless of whether it was for 4 weeks or 12 weeks. Since inflammasome activation mainly occurs in testis macrophages, we found the macrophage count decreased significantly in immature mice after 4 weeks of a HFD, and the number of macrophages was higher in mature mice after 4 week or 12 weeks of a HFD. Therefore, it has been demonstrated that the decrease of IL1β in immature mice that were fed a HFD for 4 weeks may be caused by the decrease in the number of macrophages, which induces low inflammasome activation. However, whether a HFD decreases the number of macrophages in mice-TIS testes remains to be investigated.

A few studies have found that IL1 stimulates steroidogenesis in cultured rat Leydig cells^26^. Silvina *et al*. found that IL1 treatments of purified Leydig cells did not modify basal testosterone production, while they inhibited hGC-stimulated testosterone^49^. However, more and more studies are finding that IL1β inhibits the testosterone synthesis of Leydig cell^27, 50, 51^. Here, our results indicate that IL1β inhibits testosterone synthesis of Leydig cells by down-regulating the gene expression of P450SCC and P450c17 both *vivo* and *vitro* and has no influence on Leydig cell proliferation. It is worth noting that correlation coefficient of correlation analysis on serum IL1β levels and the mRNA expression was small. It may caused by many factors can influence on the process of testosterone synthesis *in vivo*, such as TGFβ and ROS^52, 53^, the effect of IL1β on testosterone synthesis was just one part of it. As a result, our experiments indicate that the mechanism of IL1β inhibition of testosterone production may be associated with the IL1β inhibition of the steroidogenesis enzyme production. In addition, how IL1β inhibits the production of P450SCC and P450c17 needs further exploration. In summary, we provide evidence that a HFD can decrease IL1β in mice-TIS, which may induce a high testosterone level that impair fertility. However, this impairment will be recovered after stopping a HFD as long as it is stopped early. In addition, our results also reveal that a HFD impairs the reproductive system of mature mice through an increase in the IL1β level by activating NLRP3 inflammasomes and reducing testosterone levels, which increases the apoptosis of germ cells and decrease the number of offspring. Furthermore, we show that IL1β reduced testosterone biosynthesis by inhibiting the gene expression of P450SCC and P450c17. This study suggests the different effects of a HFD in mice-TIS and mice-TIS and presents a new direction for future studies on sexual development in the presence of obesity-related inflammation.

## Materials and Methods

### Establishment of high-fat diet mouse models

Four-week-old C57BL/6 mice were obtained from the DaShuo Experimental Animal Centre (Chengdu, China) and randomly divided into six groups. The mice were fed a HFD or control diet (CD) in the following different ways. Group 4H (G4H), the mice were fed a HFD for 4 weeks directly; Group 4C (G4C), the mice were fed a CD for 4 weeks; Group 8C4H (G8C4H), the mice were first fed a CD for 8 weeks and then given a HFD for 4 weeks; Group 4H8C (G4H8C), the mice were fed a HFD for 4 weeks and then given a CD for 8 weeks; Group 12H (G12H), mice were fed a HFD for 12 weeks; Group 12C (G12C), mice were fed a CD for 12 weeks; each group contained 10 mice. The control diet was a standard diet (19% casein, 0.2% L-cysteine, 29.9% corn starch, 3.3% maltodextrin, 33.2% sucrose, 4.7% cellulose, 2.4% soybean oil, 1.9% lard, 4.3% mineral mix, 0.9% vitamin mix and 0.2% choline bitartrate), whereas the high-fat diet provided contained 23.3% casein, 0.3% L-cysteine, 8.5% corn starch, 11.7% maltodextrin, 20.1% sucrose, 20.7% lard, 2.9% soybean oil, 5.2% mineral mix, 1.2% vitamin mix, 0.3% choline bitartrate and 5.8% cellulose. All mice were adapted to a 12 h light/dark cycle at 22 °C, and the animal bodyweight and food intake was weighed twice a week. Animal studies were performed in conformity with relevant guidelines and regulations and approved by the Ethics Committee of Sichuan University.

### Assessment of Sperm function parameters

The vas deferens and caudal epididymis, free of associated tissue, were obtained from experimental mice. Spermatozoa were obtained by puncturing the caudal epididymis and emptying the vas deferens into a well containing 1 ml of Tyrode’s Buffer (Sigma-Aldrich, USA). After incubated at 37 °C for 10 min, sperm motility and concentration were analyzed by computer-assisted sperm analysis (CASA) (Hamilton Thorne, USA).

### Preparation of tissue and testis homogenates

At the end of the experiment, following 12 h of starvation, the mice were anesthetized by intraperitoneal injection of chloral hydrate (5%) and were weighed using an electronic balance. Blood samples were collected from the abdominal aorta, and the serum was collected and stored at −80 °C until analysed. The testes and epididymal white adipose tissue were removed immediately, washed in cold saline, and then blotted dry with filter paper before being weighed and photographed. Additionally, after being weighed, the right testis was fixed in MDF solution^54^, and the left testis was frozen in liquid nitrogen.

For homogenates, frozen testes from each mouse were cut, weighed and homogenized on ice with saline, which contained a proteinase inhibitor at a dose of 0.1 g:1 ml. At the end of the homogenization process, the mixture was centrifuged at 4000 RPM at 4 °C for 15 min, and the supernatant was collected and stored at −80 °C.

### ELISA

An enzyme-linked immunosorbent assay (ELISA) was used to measure serum or supernatant testosterone (ENZO, ADI-900-065) and serum or testis IL1β (Develop, DL-IL1b-Mu) following the manufacturer’s instructions.

### Haematoxylin-eosin (HE) stain and Immunohistochemistry (IHC)

Each testis obtained from the mice was fixed in MDF solution for 48 h and embedded in paraffin. Then, the testes were sliced into 4-μm sections at the 1/4 and 1/2 level of the testis along the long diameter. The sections were deparaffinised and hydrated through a graded ethanol series. The slides were stained with haematoxylin-eosin for evaluation by light microscopy. Photomicrographs were obtained using a Photo Imaging System (Olympus DP20). Two level sections per testis were counted for the number of spermatogonia, spermatocytes, spermatids and Leydig cells, and six different regions of each section were chosen within random fields (400× magnification).

A sperm pellet was initially smeared on a glass slide for teratozoospermia analysis. After drying, the slide was fixed in neutral formalin for 30 min and then stained with haematoxylin for 3 min. After that, the slides were placed in eosin for 30 s covered with resin after drying. The slide was viewed under a microscope (Olympus DP20). Sperm samples obtained from G4H (n = 10) mice and G4C (n = 10) mice were analysed, and at least 200 spermatozoa in every sample were included.

For the IHC assay, endogenous peroxidase was blocked with 3% H_2_O_2_ for 15 min, and antigen retrieval was performed by using high pressure. Monoclonal rabbit anti-mouse F4/80 antibody (Abcam, ab111101) at a 1:100 dilution was used as the primary antibody and applied overnight at 4 °C. Subsequently, the tissues were incubated with a biotinylated donkey-anti-goat immunoglobulin (1:1000 dilution), and then, the slides were counterstained with haematoxylin. In each group, 18 fields (three level sections per mouse, 400× magnification) from each mouse were randomly selected to count the total number of cells in the testis interstitium and the number of F4/80-expressing cells. The positive ratio (PE) of F4/80-expressing cells in each mouse was calculated according the following formula: PE = the number of nuclei of F4/80-expressing cells/the total number of nuclei in the sections of each mouse.

### TUNEL assay

A terminal deoxynucleotidyl transferase dUTP nick end labelling (TUNEL) assay kit (Millipore, S7165) was used to measure testicular cell apoptosis in tissue sections, according to the instructions provided by the manufacturer. The slides were observed under a fluorescence microscope (Olympus, Hamburg, Germany), and the apoptotic cells were stained red, while other cells were stained blue. One hundred seminiferous tubule sections (400× magnification, Nikon E100) were randomly selected in each group, and the total number of apoptotic cells was counted. Then, the apoptosis index (AI) in each group was calculated according to the following formula: AI = total number of apoptotic cells/100.

### Cells treated with IL 1β and measurement of cell viability by MTT assay

The Leydig cell line TM3 was ordered from ATCC (GuangZhou Jennio Biotech Co., Ltd.) and cultured in DMEM/F12 supplemented with 2.5% foetal bovine serum and 5% horse serum and grown at 37 °C in an atmosphere of 5% CO_2_ in air. TM3 cells (1 × 10^4^ cells/well) were cultured in a 96-well plate under the stimulation of different concentrations of IL1β (10^−3^–10^2^ ng/ml) (Peprotech, 211-11B) for 24 h. Cell viability was assessed using an MTT assay. The colour developed was measured at 490 nm using a microplate reader (Bio-Rad, Hercules, CA, USA).

To assess the effect of IL1β on testosterone production *in vitro*, Leydig cells (1 × 10^6^) were seeded in a six-well plate and then stimulated with IL1β (10^−3^–10^2^ ng/ml) for 24 h. After that, the supernatants were collected by centrifugation at 2000 RPM for 10 min and stored at −80 °C.

### Static incubation of testicular tissue *in vitro*

To detect the direct effects of IL1β on testosterone secretion, testis tissue was incubated *in vitro* using a previously described method^55, 56^. Simply, for static incubations, the mice were killed by decapitation and blood was drawn from the abdominal aorta for serum hormone measurements. Upon death, the testes were removed immediately, decapsulated and cut into pieces of approximately equal size. Hemi-testes were incubated in 2 ml of Dulbecco’s modified Eagle’s medium (DMEM/F12; Gibco), 100 mg/ml streptomycin and 100 units/ml penicillin (Sigma) in a Dubnoff shaker (60 cycles/min) at 37 °C under an atmosphere of 5% CO_2_/95% O_2_. After pre-incubation for 2 h, the medium was replaced with medium that contained increasing doses of IL1β (Peprotech, 211-11B). Each experiment comprised seven independent hemi-testicular samples that were obtained from 6- or 10-week-old normal mice. After 24 h of incubation, the supernatants were harvested, and the testosterone concentration was measured.

### Mating assay

Male mice were mated with three normal female mice. Each group included 10 HFD-treated or not male mice. The pregnant female mice were isolated to a separate cage. The numbers of pups in the litter at the first birth were recorded. Finally, the total number of offspring was divided by the number of total mated female mice to obtain the average number of offspring for each female mouse.

### Quantitative real-time polymerase chain reaction (qRT-PCR)

To validate the expression of inflammasome mRNA and mRNA expression of testosterone-related enzymes in HFD-fed mice and CD-fed mice of immature and mature age, total RNA of Frozen testis and TM3 cell was isolated using RNAiso plus (TAKARA, Tokyo, Japan) and converted into cDNA using a PrimeScript RT reagent kit (TAKARA) according to the manufacturer’s instructions. qRT-PCR was performed with SYBR Green Master Mix (TAKARA) using an iCycler iQTM Multicolour Real-Time Detection System (BIO-RAD). The following primers were used:

NLRP3, 5′-ATTACCCGCCCGAGAAAGG-3′ and 5′-TCGCAGCAAAGATCCACACAG-3′;

ASC, 5′-CTTGTCAGGGGATGAACTCAAAA-3′ and

5′-GCCATACGACTCCAGATAGTAGC3′;

Caspase-1, 5′-AATACAACCACTCGTACACGTC-3′ and 5′-AGCTCCAACCCTCGGAGAAA-3′;

β-ACTIN; 5′-GTGACGTTGACATCCGTAAAGA-3′ and 5′-GCCGGACTCATCGTACTCC-3′;

P450SCC (CYP11A1), 5′-AGGTCCTTCAATGAGATCCCTT-3′ and 5′-TCCCTGTAAATGGGGCCATAC-3′;

3βHSD, 5′- TGGACAAAGTATTCCGACCAGA-3′ and 5′-GGCACACTTGCTTGAACACAG-3′;

P450 17Α (CYP17A1), 5′-GTCGCCTTTGCGGATAGTAGT-3′ and 5′-TGAGTTGGCTTCCTGACATATCA-3′;

17βHSD, 5′- ATTCCACGAAGTGTACTGTGC-3′ and 5′-AGGGCTTGCTCATAACCACG-3′;

STAR, 5′- CGGGTGGATGGGTCAAGTTC-3′ and 5′-GCACTTCGTCCCCGTTCTC-3′.

The relative expression levels were determined using Gene Expression Macro Version 1.1 software (BIO-RAD).

### Western blot analysis

Testes placed in lysis buffer containing RIPA buffer and protease inhibitor cocktail were dispersed with a homogenizer (Thermo Fisher Scientific, USA) on ice. Then, the lysates were centrifuged at 12,000 rpm for 15 min at 4 °C. After determination of the protein content using a Bio-Rad Protein Assay (BIO-RAD), equal amounts of extracted protein were loaded, separated via 12% SDS-PAGE and transferred to a polyvinylidene difluoride membrane (Millipore). After blocking with TBST (10 mM Tris-HCl, pH 8.0, 150 mM NaCl, 0.1% Tween-20) containing 5% skim milk for 1 h at 37 °C, the membrane was incubated with primary antibody at 4 °C overnight. The anti-NLRP3 antibody used was purchased from Abcam (ab205680), anti-Caspase1 (sc-514) and anti-β-actin was purchased from Santa Cruz Biotechnology. After incubation in primary antibody, the membrane was washed in TBST three times and then incubated in a horseradish peroxidase (HRP)-conjugated goat anti-rabbit or anti-mouse antibody (Santa Cruz Biotechnology) for 1 h at 37 °C. The membrane was developed using Immobilon Western Chemiluminescent HRP Substrate (Millipore). Densitometric ratio analyses were performed using Image J software.

### Statistical analysis

All data are expressed as the means ± standard error of the mean (S.E.M.). Statistical tests were performed using SPSS software (Version 13.0; SPSS, Inc., Chicago, IL). The means were compared using Student’s t-test or unpaired t-test with Welch’s correction to evaluate the significant differences between two groups. Multiple-group comparisons were performed via one-way analysis of variance, followed by Tukey’s post hoc test. All statistical analyses were two-sided, and P < 0.05 was considered statistically significant.

## Electronic supplementary material

Supplementary 1
