# Supplementary material for: A high-fat diet impairs reproduction by decreasing the IL1β level in mice treated at immature stage
Source: Sci Rep. 2017 Apr 3;7:567. doi: 10.1038/s41598-017-00505-0 (PMC5428732; doi:10.1038/s41598-017-00505-0)

A high-fat diet impairs reproduction by decreasing the IL1β level in mice treated at immature stage

Jie Zhang[1], kai Li[2], Miao Yuan[1], Jie Zhang[2], Guizen Huang[1], Jie Ao[2], Haoze Tan[1], Yanyan Li[2], Di Gong[2], Jun Li[2], lei Kang[1], Nini An[3], Fei Li[3], Ping Lin[2]* and Lugang Huang [1]*

Supplementary Figure legend

Supplemental Figure S1. Serum testosterone levels of control diet mice (n=10) in different ages.

Supplemental Figure S2. Correlation analysis on serum IL1β levels and the mRNA expression of STAR, P450SCC, P45017α, 3-βHSD and 17β-HSD in all experimental mice (n=40).

Supplementary Figure S3. Full-length blots for main figures corresponded to Fig. 3c and 3d.

Supplementary Figure S4. Full-length blots for main figures corresponded to Fig. 5j.

Supplementary Figure S1.

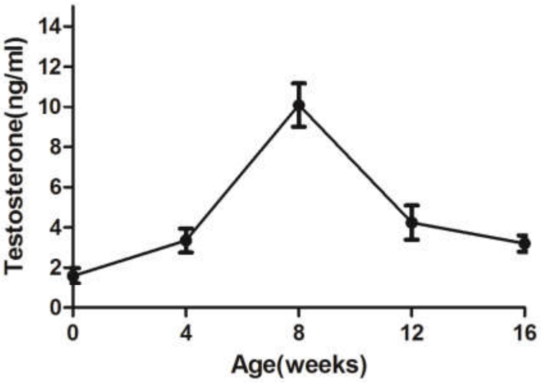

Supplementary Figure S2.

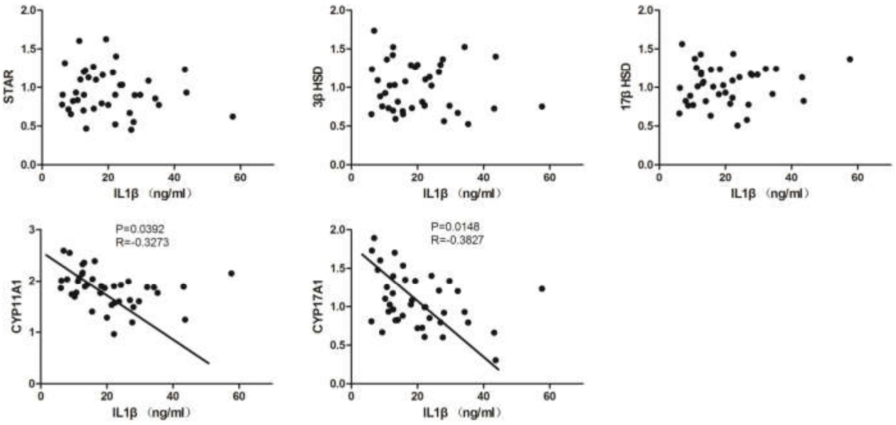

Supplementary Figure S3.

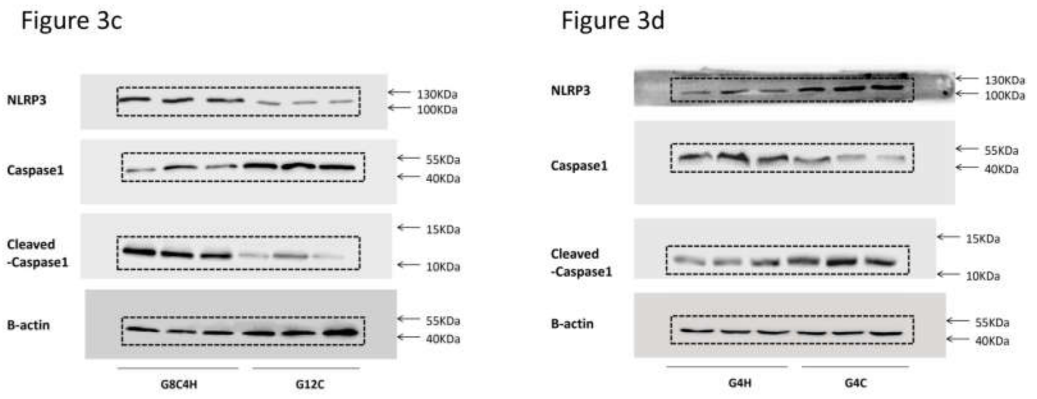

Supplementary Figure S4.

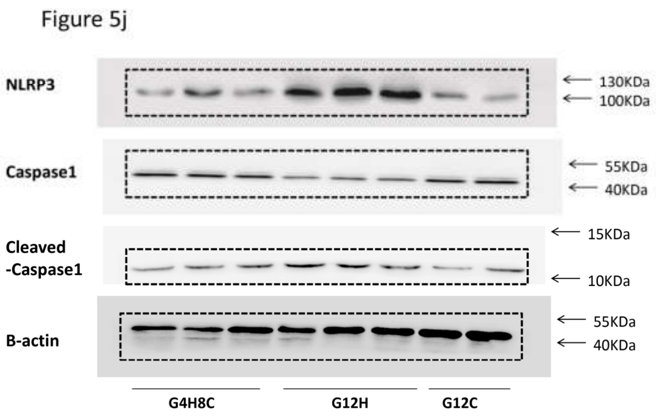

Supplement: Supplementary file 1 — Supplementary 1 [file 41598_2017_505_MOESM1_ESM.pdf]
